# Extracellular small non-coding RNA contaminants in fetal bovine serum and serum-free media

**DOI:** 10.1038/s41598-019-41772-3

**Published:** 2019-04-02

**Authors:** Bettina Mannerström, Riku O. Paananen, Ahmed G. Abu-Shahba, Jukka Moilanen, Riitta Seppänen-Kaijansinkko, Sippy Kaur

**Affiliations:** 1 Department of Oral and Maxillofacial Diseases, University of Helsinki and Helsinki University Hospital, Helsinki, Finland; 2 Helsinki Eye Lab, Ophthalmology, University of Helsinki and Helsinki University Hospital, Helsinki, Finland; 3 Department of Oral and Maxillofacial Surgery, Faculty of Dentistry, Tanta University, Tanta, Egypt

**Keywords:** miRNAs, Small RNAs

## Abstract

In the research field of extracellular vesicles (EVs), the use of fetal bovine serum (FBS) depleted of EVs for *in vitro* studies is advocated to eliminate the confounding effects of media derived EVs. EV-depleted FBS may either be prepared by ultracentrifugation or purchased commercially. Nevertheless, these preparations do not guarantee an RNA-free FBS for *in vitro* use. In this study we address the RNA contamination issue, of small non-coding (nc)RNA in vesicular or non-vesicular fractions of FBS, ultracentrifugation EV-depleted FBS, commercial EV-depleted FBS, and in our recently developed filtration based EV-depleted FBS. Commercially available serum- and xeno-free defined media were also screened for small ncRNA contamination. Our small ncRNA sequencing data showed that all EV-depleted media and commercially available defined media contained small ncRNA contaminants. Out of the different FBS preparations studied, our ultrafiltration-based method for EV depletion performed the best in depleting miRNAs. Certain miRNAs such miR-122 and miR-203a proved difficult to remove completely and were found in all media. Compared to miRNAs, other small ncRNA (snRNA, Y RNA, snoRNA, and piRNA) were difficult to eliminate from all the studied media. Additionally, our tested defined media contained miRNAs and other small ncRNAs, albeit at a much lower level than in serum preparations. Our study showed that no media is free of small ncRNA contaminants. Therefore, in order to screen for baseline RNA contamination in culturing media, RNA sequencing data should be carefully controlled by adding a media sample as a control. This should be a mandatory step before performing cell culture experiments in order to eliminate the confounding effects of media.

## Introduction

Fetal bovine serum (FBS) contains essential factors required for cell growth, metabolism, attachment, and stimulation of proliferation^1^. Thus, it is the most widely used supplement for culturing human and animal cells. One of the major concerns that has become evident recently is the presence of large amounts of extracellular vesicles (EVs) in FBS^2–4^. Secreted by most cell types, EVs are mediators of cell-to-cell communication and immune regulation^5,6^. They carry the genetic material (DNA and RNA) as well as proteins, lipids and other molecules, the transfer of which can alter the functions of the recipient cells. EVs present in FBS are co-isolated with cell-derived EVs and therefore act as contaminants that, affect the reliability of the read-out from cell culture experiments, as FBS-derived EVs are structurally and functionally similar to cell-derived EVs^7^. In addition, FBS-EVs taken up by cultured cells cause substantial physiological effects^2^. To overcome this problem, different methods are used to deplete EVs from FBS. Ultracentrifugation (UC) is commonly used, but this method removes EVs only partially^3,4,8^. Commercially available depleted FBS are produced using proprietary protocols to reach higher purity levels. Also, commercially available defined, serum-free and animal protein free (xeno-free) media developed for clinical cell therapy purposes are an alternative to the largely undefined FBS in culture media. However, we and others have shown that EV-depleted FBS produced by UC or even commercial EV-depleted FBS are not completely free of FBS-derived EVs^3,4^. Recently we have developed a simple, standardized, cost- and time-effective protocol to produce EV-depleted FBS by means of ultrafiltration (UF) for cell culture purposes that support cell viability and proliferation^9^.

In the EV research field, identifying the ‘RNA contaminants’ derived from media itself is of high importance when developing RNA biomarkers. To address this question, we undertook this study to characterize the extracellular small non-coding (nc)RNA contaminants present in FBS, EV-depleted FBS, commercially available EV-depleted FBS as well as serum- and xeno-free defined media to assess their small ncRNA content and diversity.

## Results

### EV characterization

**Figure 1:**
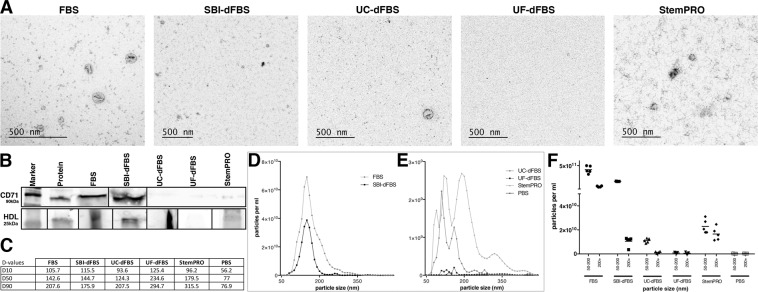
Characterization of EVs isolated by ultracentrifugation. Transmission electron microscopy analysis revealed the presence of vesicles in FBS, UC-dFBS and SBI-dFBS. StemPRO contained scarce vesicle-like structures and abundant protein aggregates, while no vesicles could be detected in UF-dFBS (**A**). Western blotting (**B**) displayed strong expression of CD71 in FBS and SBI-dFBS, no expression in UC-dFBS and UF-dFBS, whereas low expression was detected in StemPRO. Expression of HDL was seen in all samples except UF-dFBS. Nano tracking analysis (**C**–**F**) indicated that UF-dFBS had the lowest particle count. Particle distribution (**C–F**). (**C**) D10 = 10% of particles are below the size indicated as D10, D50 = 50% of particles are below the size indicated as D50, D90 = 90% of particles are below the size indicated as D90. Median particle size in FBS, dFBS and UC-FBS are in a similar range, while UF-dFBS is considerably different. (**D,E**) Particle concentration and distribution. FBS and SBI-dFBS showed similar particle concentrations. (**F**) X-axis; particle size (nm) in two size ranges; 50–200 nm and 200 + nm.

EVs were isolated from FBS, ultracentrifugation EV-depleted FBS (UC-dFBS), ultrafiltration EV depleted FBS (UF-dFBS), commercial depleted FBS (SBI-dFBS) and commercial serum- and xeno-free media (StemPRO) by ultracentrifugation (Table 1). EVs were further characterized by nano tracking analysis (NTA), transmission electron microscope (TEM) and western blotting (WB). Based on TEM analysis, while no vesicles could be detected in UF-dFBS, vesicles structures and protein aggregates were detected in regular FBS, UC-dFBS and SBI-dFBS. StemPRO displayed scarce vesicle-like structures and large protein aggregates (Fig. 1A). For western blotting (WB) analysis, bovine specific EV markers are lacking and species cross reactivity for bovine is rarely reported. Anti-tetraspanin antibodies were not used as it is difficult to estimate whether these antibodies specific for mouse or human samples recognize the bovine form. We therefore selected anti-transferrin receptor/CD71 H68.4 monoclonal antibody, which is abundant in serum-derived material, suitable for both mouse and bovine proteins and also recognizes a transmembrane protein, therefore its detection confirms that EVs are analyzed^10,11^. WB results indicated a strong CD71 band in FBS and SBI-dFBS, whereas no bands were detected in UC-dFBS and UF-dFBS. Faint band of CD71 protein was detected in StemPRO (Fig. 1B). To address the contamination issue, we also performed an additional WB analysis (Supplementary Fig. 1) with HDL (ApoA1) to assess the presence of non-EV bound miRNAs in our samples. Since the antibody was specific for human proteins, species cross-reactivity was analyzed running human plasma samples in parallel with FBS samples. No primary antibody control was used to assess if any non-specific binding or false positives may be due to non-specific binding of the secondary antibody. Pure human HDL was added as a positive control. Thereby confirming the reliability of the results.

**Table 1:** Suppliers and pricing information of culture media used for small ncRNA sequencing.

Acronym	Culture media	Basal media	Serum manufacturer	Serum content	Supplementation	Price/50 ml (excl. VAT)
FBS-1	FBS-1 media	DMEM/F12 + Glutamax cat# 31331093	Sigma, ref. 10270106, lot. 42F8554K	10% FBS	1% pen-strep	4 eur
FBS-2	FBS-2 media	DMEM/F12 + Glutamax cat# 31331093	Gibco, ThermoFisher cat# 10270106 lot. 42G8468K	10% FBS	1% pen-strep	4 eur
UC-dFBS-1	Ultracentrifugation EV-depleted FBS media	DMEM/F12 + Glutamax cat# 31331093	Sigma, ref. 10270106, lot. 42F8554K	10% UC-dFBS	1% pen-strep	36 eur
UC-dFBS-2	Ultracentrifugation EV-depleted FBS media	DMEM/F12 + Glutamax cat# 31331093	Gibco, ThermoFisher cat# 10270106 lot. 42G8468K	10% UC-dFBS	1% pen-strep	35 eur
UF-dFBS-1	Ultrafiltration EV-depleted FBS media	DMEM/F12 + Glutamax cat# 31331093	Sigma, ref. 10270106, lot. 42F8554K	10% UF-dFBS	1% pen-strep	53 eur
UF-dFBS-2	Ultrafiltration EV-depleted FBS media	DMEM/F12 + Glutamax cat# 31331093	Gibco, ThermoFisher cat# 10270106 lot. 42G8468K	10% UF-dFBS	1% pen-strep	52 eur
SBI-dFBS-1	Commercial Exosome depleted FBS media	DMEM/F12 + Glutamax cat# 31331093	System Biosciences, ref. EXO-FBS-50A-1 lot.170501-001	10% SBI-dFBS	1% pen-strep	39 eur
SBI-dFBS-2	Commercial Exosome depleted FBS media	DMEM/F12 + Glutamax cat# 31331093	System Biosciences, ref. EXO-FBS-50A-1 lot.170718-001	10% SBI-dFBS	1% pen-strep	39 eur
StemPRO	Commercial StemPro MSC Serum free medium	StemPro® MSC SFM Basal Medium	Gibco, Thermo Fisher, A1067501	—	0.3% pen-strep, 1% StemPro MSC CFM Xenofree supplement, GlutaMAX™ Supplement	37eur^a^

Pricing information include consumables and supplementation needed for preparation of 50 ml of complete media.

^a^culture vessels need to be coated with CELLstart™ Substrate (Thermo Fisher cat# A1014201) 1:50 dilution 3.6 eur/ml.

Characterization of the EV samples by NTA supported the WB results (Fig. 1C–F). The NTA results showed that as compared to other FBS and SBI-dFBS (Fig. 1D), the number of particles in StemPRO and UC-dFBS was lower, while UF-dFBS had a very low particle count (Fig. 1E). Regular FBS and SBI-dFBS had similar particle number (e^11^) and particle distribution (Fig. 1C). Together, these results indicate that UF-dFBS has a low particle and protein content, potentially being nearly EV-free. Next, we wanted to address the small ncRNA content in these preparations.

### General mapping results of small ncRNA sequencing

**Figure 2:**
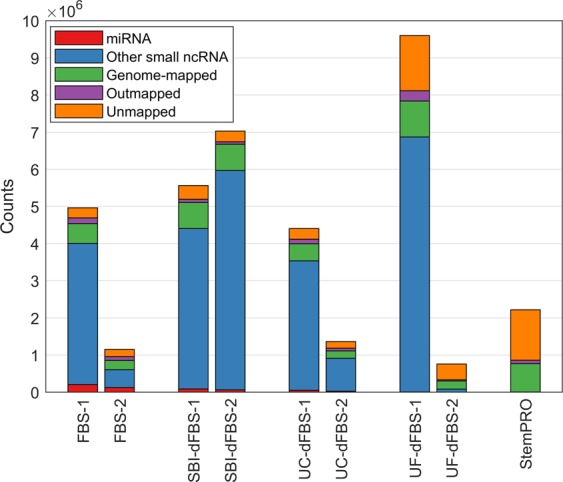
Overview of sequencing data and mapping to human reference genome. Number of counts mapped to miRNA, other small ncRNA (tRNA fragments, snRNA, Y RNA, snoRNA or piRNA) for each sample are shown. Genome-mapped refers to reads that aligned to the human reference genome outside small ncRNA loci. Outmapped counts were mapped to adapters, rRNA, mtRNA or polyA/polyC homopolymers.

Small ncRNA sequencing was performed on FBS containing media (regular FBS and EV-depleted FBS) and serum- and xeno-free media (Table 1). RNA sequencing was not feasible on basal media and supplements due to low RNA yield. Mapping of our sequencing data to human reference genome showed that 0.8 to 9.6 million reads per sample were obtained in the sequencing experiments (Fig. 2). No clear pattern in raw read counts was observed with the different depletion treatments, but samples based on FBS-2 (dFBS-UC-2 and dFBS-UF-2) showed 3- to 10-fold lower raw reads than their FBS-1-based counterparts.

### Mapping of small ncRNA sequencing reads to bovine and human genome displayed indistinguishable miRNA reads and non-overlapping small ncRNA reads between the two species

**Figure 3:**
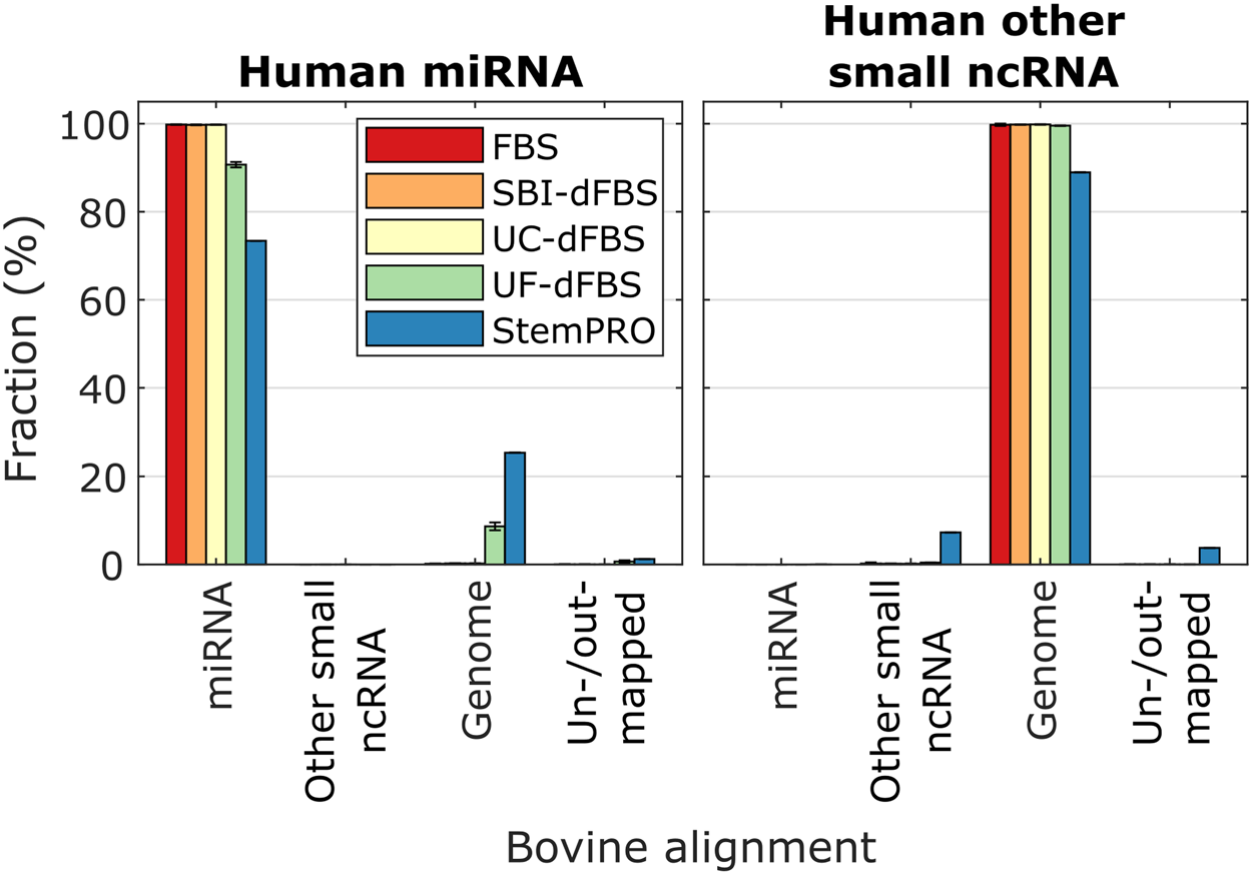
Overlap in alignment to human and bovine reference genomes. Reads aligned human small ncRNAs (miRNA and other ncRNA) were further mapped to a bovine reference genome to estimate the likelihood that these RNAs are of bovine origin. Genome refers to reads that aligned to the bovine reference genome outside small ncRNA loci. Un/outmapped reads either failed to map or were mapped to adapters, rRNA, mtRNA or polyA/polyC homopolymers.

Small ncRNA sequencing reads were mapped to both human and bovine genome to detect the extent of species-specificity and overlap between the species. Our analysis showed (Fig. 3) that human miRNAs in the tested culture media mapped almost completely (90–100%) to bovine miRNAs, which is also consistent with previous report^4^. The similarity between bovine and human miRNAs makes distinguishing bovine miRNAs from human miRNA difficult, which may be a confounding factor when assessing miRNA derived from cell culture experiments. In StemPRO medium the overlap was only 73%, which suggests that a much more significant proportion of these reads may not have originated from miRNA at all, but may rather be experimental noise, suggesting that the actual miRNA content of the defined media was very low. In contrast to miRNAs, other human small ncRNAs were also mapped to a high degree (90–100%) to the bovine genome. The reason for this is that the database for bovine small ncRNAs did not contain any tRNA fragments, which made up most of the human small ncRNA alignments. Therefore, almost all human small ncRNA reads were classified as mapping to the bovine genome instead of mapping to small ncRNAs.

### Vesicular and non-vesicular derived small ncRNA contaminants in FBS, dFBS and commercial media

**Figure 4:**
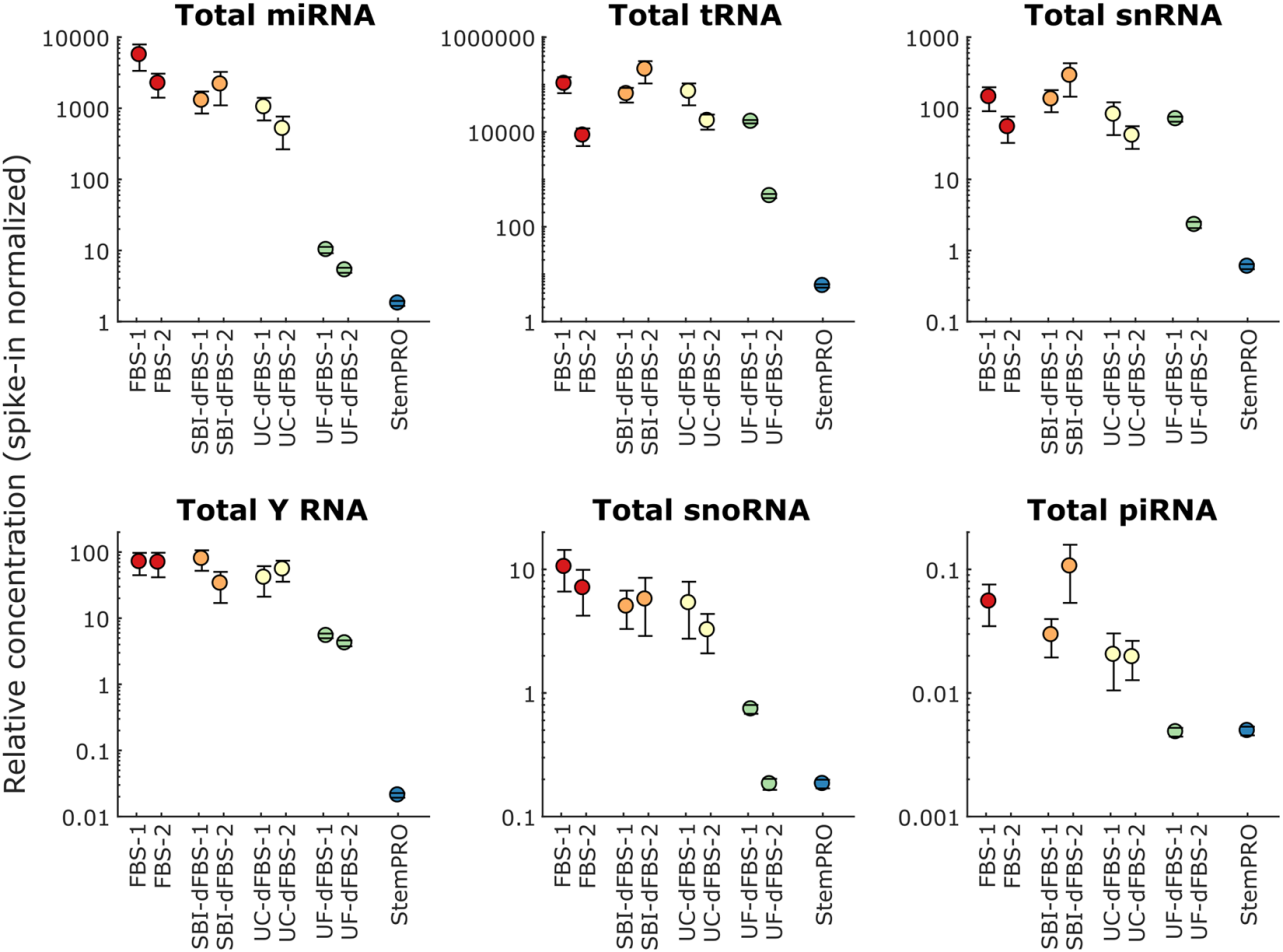
Spike-in normalized concentration of different small ncRNA in studied media. Total miRNA, tRNA, snRNA, Y RNA, snoRNA, and piRNA concentration is shown for each media sample. Error bars depict standard deviation of the spike-in fit.

We further characterized the small ncRNA composition of FBS media preparations. As a quality control, serum and xeno-free media (StemPRO) was included in the study and evaluated for RNA contamination, as this medium should not contain any animal derived components. To facilitate comparison between different media types, total miRNA and other small ncRNA levels were normalized according to the external spike-in levels. As shown in the Fig. 4, StemPRO contained small amounts of small ncRNA, source of which is not clearly known. As shown in Fig. 4, considerable variation was observed in RNA content between different media types and manufacturers. In untreated FBS, most (93%) of all small ncRNA counts were mapped to tRNA, whereas 6.5% were mapped to miRNA. Other types of small ncRNA (snRNA, Y RNA, snoRNA, piRNA) were detected in only small amounts (<0.2%). As expected, highest levels of miRNA were detected in untreated FBS, whereas commercial SBI-dFBS had 60 ± 20% lower total miRNA counts. Ultracentrifugation and ultrafiltration lowered the total miRNA content by 80 ± 9% and 99.8 ± 0.1%, respectively. Commercial serum-free medium StemPRO had 99.95 ± 0.01% lower total miRNA content than FBS. For tRNA and snRNA, a similar clear trend was not observed, and depleted media had similar levels of tRNA and snRNA as untreated FBS, although large variation between individual samples was observed (Fig. 4). For Y RNAs and snoRNAs, approximately 10-fold reduction was observed in UF-dFBS compared to other FBS-based media. StemPRO had very low levels of any kind of small ncRNAs. Full list of spike-in normalized miRNA and other small ncRNA counts are listed in Supplementary 1. It should be noted that since the spike-ins used were miRNA-sized (20–24 nt), the normalization is likely to be more accurate in the case of miRNAs than other small ncRNA, which were mostly longer (~30 nt).

**Figure 5:**
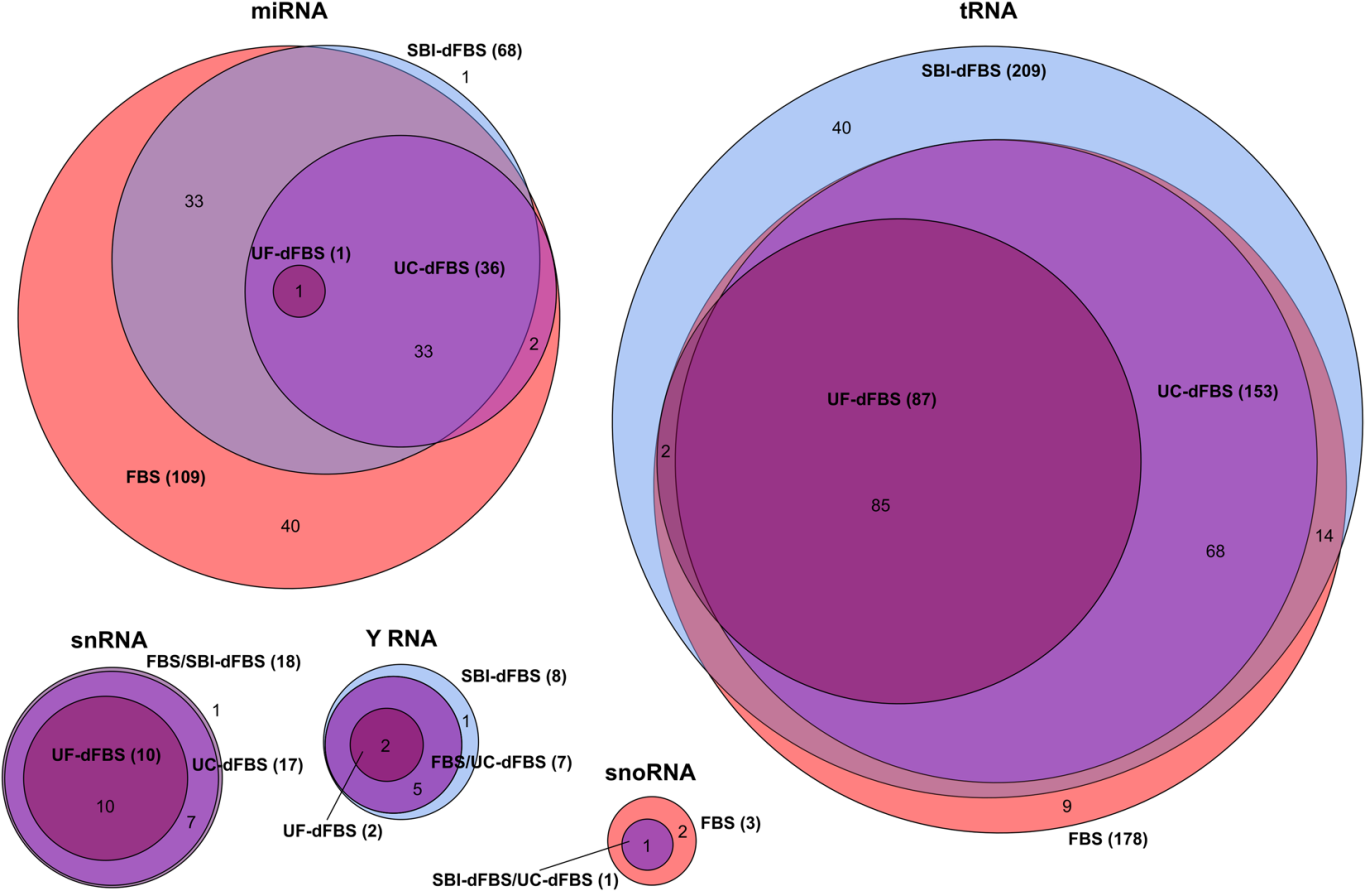
Euler diagrams showing the relationships of the small ncRNA sets detected in different media types. RNAs detected at above 1 spike-in normalized count level (moderate to high abundance) were included in the diagrams. Total number of different RNAs per media type are shown in parenthesis. No small ncRNAs were detected above this level in the StemPRO sample.

For depicting the overlap of small ncRNAs in the different media, Euler diagrams were created (Fig. 5). Our data showed that almost all of the detected miRNAs were found in FBS (109 miRNA detected). Only one miRNA, miR-133a-3p, was not detected in FBS or FBS-derived media, but only in commercial SBI-dFBS media. As expected, all the miRNAs in depleted media were also found in untreated FBS. EV depletion techniques resulted in lower number of miRNAs detected above threshold: 68 for SBI-dFBS, 36 for UC-dFBS and only 1 for UF-dFBS. Similar pattern was observed for other small ncRNA, with all the RNAs detected in FBS-based depleted media also present in untreated FBS and 40 unique tRNA fragments and one Y RNA found in SBI-dFBS media. Less effective depletion was observed with tRNA, snRNA, Y RNA and snoRNA, with UF-dFBS still containing 87 out of the 178 tRNA, 10 out of 18 snRNA and 2 out of 8 Y RNA detected in untreated FBS.

### Comparative analysis of RNA depletion efficiency

**Figure 6:**
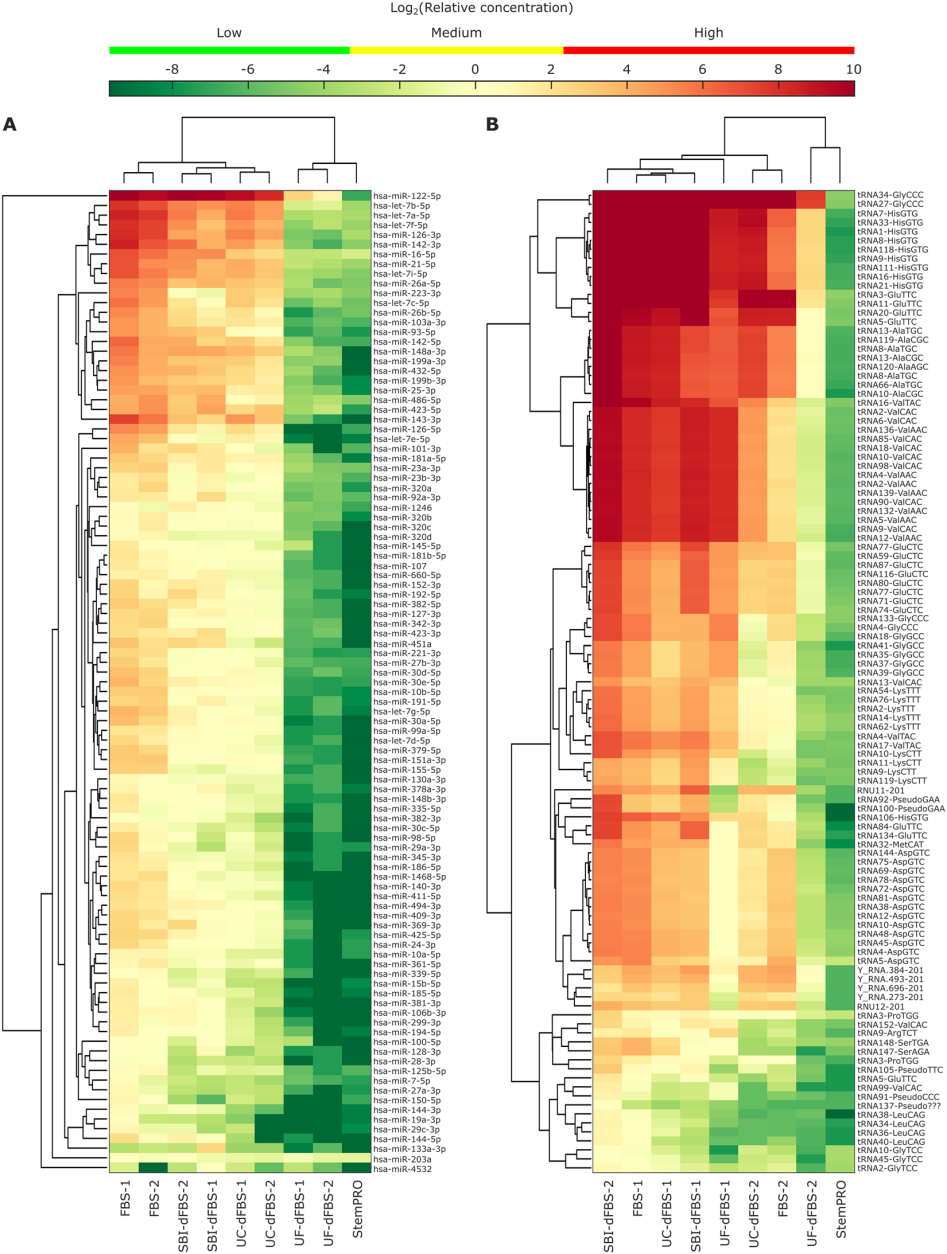
Unsupervised clustering analysis of the most abundant miRNAs (**A**) and other small ncRNAs (**B**). Top 60 most abundant miRNAs and top 40 most abundant other small ncRNAs in any samples were selected for the clustering analysis, resulting in 101 miRNAs and 109 other small ncRNAs. Heatmaps are showing the relative concentration of each RNA, normalized using the spike-in miRNAs. Medium level refers to the concentration range of spike-ins, whereas low level is lower than any of the spike-ins and high level is higher than any of the spike-ins used.

Subsequently, we evaluated the RNA depletion efficiency of different EV depletion methods. Unsupervised clustering analysis (Fig. 6A) showed that in the case of miRNAs, sample types clustered together, and a uniform decrease in miRNA levels across almost all miRNAs was observed with the depletion treatments. hsa-miR-122–5p was clearly the most abundant miRNA in FBS-based media. Furthermore, we also investigated how many of the top 20 most commonly reported EV miRNAs in the literature are present in different media preparations (Supplementary 2, table B). Several of the commonly reported miRNA in EVs like hsa-miR-21-5p, hsa-miR-451a, hsa-miR-16-5p, hsa-let-7a, hsa-let-7b and hsa-miR-93-5p has-miR-93-5p were found in high levels even in some of the depleted media preparations. In contrast, clustering based on other small ncRNAs (Fig. 6B) occurred based on FBS supplier (1 and 2; Table 1) rather than sample type. No clear reduction in tRNAs was observed by the depletion treatments. This indicated that tRNA, which were the most abundant type of small ncRNA detected in the media samples, was not effectively removed with the depletion treatments. However, a large difference in tRNA levels was observed between the two FBS suppliers tested.

### Enrichment of mir-203a in all media

The sequencing data clearly indicated ineffective depletion of RNA in dFBS, commercially available EV-depleted FBS and defined media, and the efficiency of miRNA depletion in these depleted media is unclear. To address this, we focused on specific miRNAs and traced their levels in the different media preparations. Based on the clustering analysis, two groups of miRNAs were selected for further analysis; miRNAs strongly affected by depletion methods and miRNAs weakly affected by depletion methods (Supplementary 2, figure). As can be seen, there is little difference between the groups, indicating that almost all the miRNAs are similarly affected by the depletion methods. A notable exception is hsa-miR-203a, which was present in StemPRO and appeared to be unaffected by all of the depletion methods.

## Discussion

Interference by FBS derived RNA (vesicular or non-vesicular) in the *in vitro* studies has recently become evident^2–4^, Therefore, reliability of the results depends on the complete elimination of serum derived RNA. Until now, little attention has been paid to characterizing these RNA contaminants. Ultracentrifugation, the standard method used for EV depletion, is unable to provide EV-free FBS. Moreover, commercially available EV-depleted FBS are also not completely EV/RNA-free^9^. Therefore, we recently developed an ultrafiltration-based protocol to generate EV-free serum. Consequently, we undertook this study to define the small ncRNA contamination present in different depleted FBS preparations, including our ultrafiltration-based EV- depleted FBS, commercially available EV-depleted FBS and serum- and xeno-free media.

It is well known that the specificity of isolating EVs is low for both ultracentrifugation and other EV isolation methods. Various studies have demonstrated that EV preparations derived using these methods contain small amounts of non-EV soluble components, which might carry RNA as well^12–14^. EV isolation and RNA sequencing was done according to the stringent workflow of the Qiagen services. Furthermore, qPCR was performed on our media samples with endogenous miRNAs (miR-451a, miR-103a-3p, miR-191-5p, miR-23a-3p, miR-30c-5p, mir-23a-mir451a and 3 spike in controls; UniSp6, UniSp-101, and UniSp-100). mir-451a, used as an EV marker^15^, was clearly expressed in our samples, indicating that miRNAs analyzed are EV related. Despite using a stringent protocol for isolating EV-related RNA for RNA sequencing, we still could not rule out the possibility of presence of non-vesicular RNA in our data. As the genuine association of sequenced RNA with EVs was not demonstrated (by using differential nuclease/protease and detergent treatments, for instance) in our study due to technical difficulties, it is more likely that our samples contained both “cell-free RNA” (non-vesicular RNA) and “EV-bound RNA” (vesicular RNA). Therefore we prefer to use the term “vesicular or non-vesicular fractions” in this study.

A vast majority of miRNAs (90–95%) are estimated to be non-vesicular^16,17^. Based on information retrieved from the literature, similar trend was observed for our miRNA data (Supplementary 2, table A). A possible source of miRNA contamination could be the circulating miRNA bound to various carriers, including Argonaute2 complexes, albumin and lipoproteins. Since high density lipoprotein (HDL) HDL-miRNA transport appears to be more robust than low density lipoprotein (LDL)^18^, we focused on analyzing HDL (ApoA1) in our samples. As expected, all the samples except UF-dFBS showed positive expression of HDL, indicating that none of the EV-depleted media are EV or lipoprotein free. These HDL-associated miRNA, could be considered as one of the source of miRNA contamination.

Our data was in agreement with Wei *et al*.^4^, where 100% overlap of RNA sequencing between bovine and human genome was observed. These results indicate, that media selected for cell culture experiments should be chosen carefully, prioritizing EV/RNA-free media. Screening of contaminants from the culture media should be a routine procedure in order to establish the baseline level of RNAs as a control to minimize the confounding effect of media. To highlight the importance of these findings, we collected the most commonly reported miRNAs in cell-culture-based EV studies (Supplemental 2, table B) and observed their levels in different media types. Majority (15 out of 20) of the most commonly reported miRNAs were found in moderate to high levels, while 5 out of 20 were found in very high levels.

UF-dFBS showed an exceptional performance in depleting miRNAs compared to UC-dFBS and SBI-dFBS. Surprisingly, miRNA levels in commercially available SBI-dFBS were slightly higher than UC-dFBS, and some miRNA, such as miR-122-5p and miR-203a were present in at least moderate levels in all dFBS. miR-122-5p was also recently reported to be present in abundant amounts in FBS and dFBS, which indicates that certain miRNAs are difficult to remove completely. In the case of miR-122-5p, this is due to its high abundance in FBS. Even though more than 99% is removed, moderate levels are still left in UF-dFBS. Liver specific miR-122 is exclusively non-vesicular, with key roles in hepatocyte growth, metabolism, and homeostasis^19^. miR-122 is also associated with human liver cancer metastasis and lung cancer^20^.

It is difficult to explain the inefficiency of removing miR-203a. It was detected mainly as short, 16-nt inserts, and could potentially be an experimental artifact, which would explain its presence in all the samples, even in StemPRO. It has been reported that miR-203 has an effect on many properties of cells in culture, e.g. as an inhibitor of ‘stemness’ of mammary stem cells and pluripotent stem cells and it may also have anti-tumorigenic activity in cancer stem cell populations^21–24^.

One striking observation in our study is that other small ncRNA (snRNA, Y RNA, snoRNA, and piRNA) contaminants are clearly more difficult to eliminate, even with our UF-dFBS (Figs 4 and 6). Despite being fully defined, StemPRO media was not completely RNA free, small traces of both miRNA and small ncRNA were detected. This is an important point to consider, as these GMP produced media are intended for clinical stem cell therapy.

Regardless of its source, presence of RNA (vesicular/non-vesicular) in all the tested media should still be considered contamination, which needs careful consideration for further applications. While there is no data readily available, we speculate that possible source of the RNA in these media could be from the recombinant proteins such as human serum albumin, essential amino acids, or vitamin constituents^25,26^.

It is likely that RNA detected in our study could have originated from the different components of media supplements. In the future additional studies are needed to formulate an RNA free culture media, where all media components should be precisely screened. This will provide the details about the function and origin of the different RNA components in media.

In conclusion, our results highlight the need and importance of screening of EV- and RNA-free media for the *in vitro* studies in the EV field, specifically related to the investigation of RNA biomarkers. Furthermore, functional impact of these contaminant RNA in recipient cells need to be addressed to understand their biological roles.

## Materials and Methods

### FBS

FBS was obtained from two different suppliers, product details are described in Table 1. FBS obtained from Sigma was referred as FBS-1 and from Gibco as FBS-2.

### EV-depleted FBS preparations

**Figure 7:**
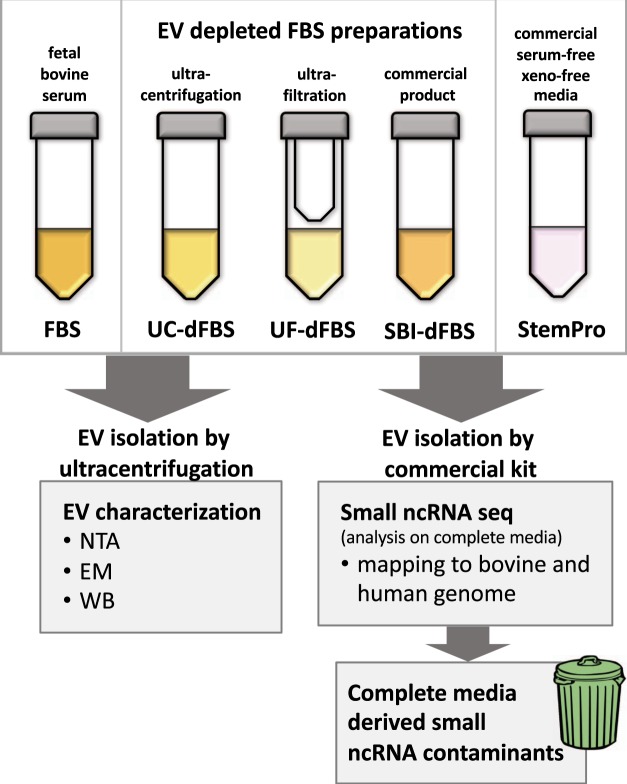
Flowchart of the study design. EV depleted FBS was prepared by ultracentrifugation, ultrafiltration or purchased from commercial supplier. Detailed protocol for the preparation of EV depleted FBS is described in materials and methods section. EVs extracted by ultracentrifugation from 10 ml of FBS, EV depleted FBS and commercial defined media were characterized by NTA, TEM and WB. For small ncRNA sequencing, EVs and RNA were extracted using exoRNeasy Serum/Plasma Maxi Kit (Qiagen) from 3.5 ml of media containing 10% depleted FBS. Reads were mapped to both bovine and human genome and media derived small ncRNA were identified.

EV-depleted FBS was prepared from both FBS-1 and FBS-2. Ultracentrifugation and ultrafiltration were used to deplete EVs from FBS. The preparation of UC-dFBS and UF-dFBS from regular FBS was done according Kornilov *et al*.^9^. Briefly, UC-dFBS was prepared by 19 hours UC of regular FBS at 26,000 rpm (121 896 *g*_max_) with SW28 rotor (k-factor 284.7, Beckmann-Coulter). Light-colored top layer of supernatant was filtered with a 0.22 µm filter (Millipore Stericup-GP, 0.22 µm, polyethersulfone filter). UF-dFBS was prepared by centrifuging regular FBS for 55 min at 3,000 *g* in Amicon ultra-15 centrifugal filters (ref: UFC910024, 100 kDa Merck Millipore Ltd., Tullagreen, Carrigtwohill, Co. Cork, Ireland) and collecting of flow-through. FBS-1, UC-dFBS-1 and UF-dFBS-1 refer to FBS obtained from Sigma and FBS-2, UC-dFBS-2 and UF-dFBS-2 refer to FBS obtained from Gibco (Table 1). Commercially available EV-depleted FBS (Exo-FBS™, System Biosciences, EXO-FBS, Mountain View, CA, USA), termed as SBI-dFBS, was used as a control (Table 1). Flowchart of the study design is illustrated in Fig. 7.

### Defined media

StemPRO^®^ MSC SFM medium, a fully defined media formulation for culturing mesenchymal stem cells, was used as a serum-free, xeno-free culture media control. For media supplementation, see Table 1.

### EV isolation from FBS preparations and defined media

EV characterization was performed on EVs isolated by UC due to technical issues related to using exoRNeasy Serum/Plasma Maxi Kit (Qiagen) used for the sequencing study. This issue has been described in our previous article^27^ Briefly, EVs were isolated from 10 ml of FBS, UC-dFBS, UF-dFBS, SBI-dFBS, and StemPRO by ultracentrifugation for 2 hours at 26,000 rpm (121 896 gmax, SW28 rotor, 4 °C) to collect an EV pellet, which was washed with filtered DPBS (0.1 μm filter) and stored in Protein LoBind microcentrifuge tubes (Eppendorf) at −80 °C. For small ncRNA sequencing, EVs and RNA were isolated from 3.5 ml of FBS, UC-dFBS, UF-dFBS, SBI-dFBS, and StemPRO by exoRNeasy Serum/Plasma Maxi Kit (Qiagen).

### Nanoparticle tracking analysis

The number and size distribution of particles in EV samples were analysed using NTA (Nanosight LM14, NanoSight Technology, Salisbury, U.K., http://www.malvern.com) as described previously^9^. Briefly, isolated EVs were diluted in filtered (0.1 µm) DPBS to obtain the optimal detection concentration of 10^6^–10^9^ particles/ml, and triplicate videos were recorded. The data was analysed using NTA software^28^.

### Transmission electron microscopy

Particle morphology was examined using TEM as described previously^9,28^. Briefly, isolated EVs were diluted in filtered (0.1 µm) DPBS, loaded into 200 mesh copper grids and negatively stained with neutral uranyl acetate and embedded in methyl cellulose uranyl acetate mixture.

### Western blotting

WB was performed as described previously^9^ using primary antibodies against anti-transferrin receptor/CD71-H68.4 (#13-6800, Thermofisher Scientific) at 1:1000 dilution and ApoA1^29,30^ (kind gift from Matti Jauhiainen) at 1:2000 EVs isolated by ultracentrifugation from equal volumes (20 ml) of each sample were loaded to gels. As a control, protein from adipose tissue derived mesenchymal stem cell lysates were used. Samples were denatured at 95 °C for 5 min in reducing Laemmli sample buffer, separated using Mini-PROTEAN^®^ TGX^™^ 12% gradient SDS-PAGE gel (#456-1043, Bio-Rad) with BlueSTAR Prestained Protein Marker (#MWP03, Nippon Genetics Europe GmbH) as a standard. Running conditions were 150 V for 60 minutes. Blotting involved semi-dry transfer of proteins on nitrocellulose membranes, 0.2 µm (#162-0112, Bio-Rad), using 40 mA per gel for 60 minutes. Blocking and antibody incubations were performed in Odyssey blocking buffer (PBS) (#927-40000, LI-COR) with and without 0.1% Tween-20, respectively. After primary antibody overnight incubation at +4 °C, membranes were washed 4 × 5 minutes in TBS-T and probed with secondary IRDye® 800CW Goat (#925-32210, LI-COR) at 1: 15,000 and goat anti-rabbit (#925-32210, LI-COR) at 1:10000 for 1 hour at room temperature (RT). After incubation, membranes were washed 4 × 5 minutes in TBS-T at RT and briefly rinsed with PBS 1X, then imaged on an Odyssey FC Imager (LI-COR).

### Small non-coding RNA (small ncRNA) sequencing

Small ncRNA sequencing was performed by Qiagen (Qiagen, Hilden, Germany). EVs and RNA were isolated using exoRNeasy Serum/Plasma Maxi Kit. EVs were isolated from 3.5 ml of media followed by RNA isolation protocol optimized for serum/plasma (no carrier RNA was added). Library preparation was done using the QIAseq miRNA Library Kit. Out of 12 µl of isolated RNA, 5 µl was converted into microRNA NGS libraries. Adapters containing 12nt-long unique molecular indices (UMIs) were ligated to the RNA. Then RNA was converted to cDNA. The cDNA was amplified using PCR (22 cycles) and during the PCR indices were added. After PCR the samples were purified. Library preparation quality control was performed using either Bioanalyzer 2100 (Agilent) or TapeStation 4200 (Agilent). Based on the quality of the inserts and the concentration measurements the libraries were pooled in equimolar ratios. The library pools were quantified using the qPCR ExiSEQ LNA™ Quant kit (Exiqon). The library pools were then sequenced on a NextSeq500 sequencing instrument according to the manufacturer instructions using 1 × 75 bp reads. Raw data was demultiplexed and FASTQ files for each sample were generated using the bcl2fastq software (Illumina Inc.).

### Data analysis

#### UMI correction and trimming

To correct PCR bias with UMI information, raw reads were processed as follows: First, cutadapt 1.11^31^ was used on each raw read with the adapter sequence to acquire information about the presence of adapter. Only reads that fulfilled the following criteria were kept: (1) read contained adapters, (2) read had at least 16nt insert sequence length, and (3) read had at least 10nt-long UMI sequence. Second, insert sequences with incomplete UMI sequence were extracted as partial-UMI reads. Third, all unique insert + UMI combinations were identified from reads with full-length UMI and collapsed into a single read. Fourth, collapsed full-length UMI reads and partial-UMI reads were combined. After UMI-correction, reads were trimmed using cutadapt output and an in-house script.

#### Mapping

Reads were mapped using Bowtie 2 (2.2.2)^32^ as follows: First, reads mapped to spike-ins or outmapped (mapped to adapter sequences, polyA and polyC homopolymers, or abundant ribosomal or mitochondrial RNA sequences) were filtered out. Perfect match to the reference sequence was required. Second, reads were aligned to mature sequences of miRBase 20, requiring perfect matches. Third, unmapped reads were mapped to either to human GRCh37 or bovine UMD3 reference genome, allowing one mismatch in the first 32 bases of the read. Reads aligned to known miRNA loci in the genome were combined with the miRBase-mapped reads and reads aligned to small ncRNA reqions in the Exiqon small ncRNA database were classified as small ncRNA. Reads mapping to the reference genome outside the miRNA or small ncRNA regions were classified as genome-mapped. No indels were allowed in any of the mapping steps.

#### Statistical analysis

Read counts were normalized using the external spike-ins (UniSp100–UniSp151) that were present in three relative concentrations of 0.1 (low), 1 (moderate) and 5 (high), reflecting the expected range of miRNAs in the samples. Linear functions with intercept fixed at zero were fitted to the spike-in concentration versus observed counts in each sample. The linear fit was used calculate an estimated relative concentration for each RNA. For the clustering analysis, only RNAs with moderate (>1) spike-in normalized counts in at least one sample were included. Clustering analysis was performed on lists of miRNAs and small ncRNAs, which included the top 60 most abundant miRNAs and 40 most abundant small ncRNAs in all samples. Euclidean distance metric and average linkage were used in the unsupervised clustering analysis. Euler diagrams were created using a threshold of 1 spike-in normalized counts (moderate to high abundance) using the eulerr-package in R.

#### Identifying commonly reported miRNAs

To identify most commonly reported EV miRNAs in cell culture studies, we used EVpedia^33^ and miRandola^34^ databases. Studies using human cultured cells were included. In miRandola, only studies with exosome or microvesicle material were included. This resulted in 19 included studies in EVpedia and 10 studies in miRandola. Three studies were reported in both databases, resulting in a total of 26 studies (Supplementary 2, table B).

## Supplementary information

Supplemental figure 1

Supplementary 1

Supplementary 2

## Data Availability

The RNA sequencing data has been deposited to the GEO (accession number GSE120594).
