# Publisher Correction: Extracellular small non-coding RNA contaminants in fetal bovine serum and serum-free media

**DOI:** 10.1038/s41598-020-57848-4

**Published:** 2020-01-23

**Authors:** Bettina Mannerström, Riku O. Paananen, Ahmed G. Abu-Shahba, Jukka Moilanen, Riitta Seppänen-Kaijansinkko, Sippy Kaur

**Affiliations:** 1 Department of Oral and Maxillofacial Diseases, University of Helsinki and Helsinki University Hospital, Helsinki, Finland; 2 Helsinki Eye Lab, Ophthalmology, University of Helsinki and Helsinki University Hospital, Helsinki, Finland; 3 Department of Oral and Maxillofacial Surgery, Faculty of Dentistry, Tanta University, Tanta, Egypt

Correction to: *Scientific Reports* 10.1038/s41598-019-41772-3, published online 02 April 2019

The original version of this Article was published with incorrect versions of Figure 1 and Supplemental figure 1 due to a technical error.

These errors have now been corrected in the PDF and HTML versions of the Article, and in the accompanying Supplementary Information.

