# Supplementary material for: Extracellular small non-coding RNA contaminants in fetal bovine serum and serum-free media
Source: Sci Rep. 2019 Apr 2;9:5538. doi: 10.1038/s41598-019-41772-3 (PMC6445286; doi:10.1038/s41598-019-41772-3)

Extracellular small non-coding RNA contaminants in fetal bovine serum and serum-free media

Bettina Mannerström, Riku O Paananen, Ahmed AG Abu-Shahba, Jukka Moilanen, Riitta Seppänen-Kaijansinkko, Sippy Kaur

Supplemental Figure 1 Original western blot images for all samples. Samples unmarked are unrelated to this project.

CD71

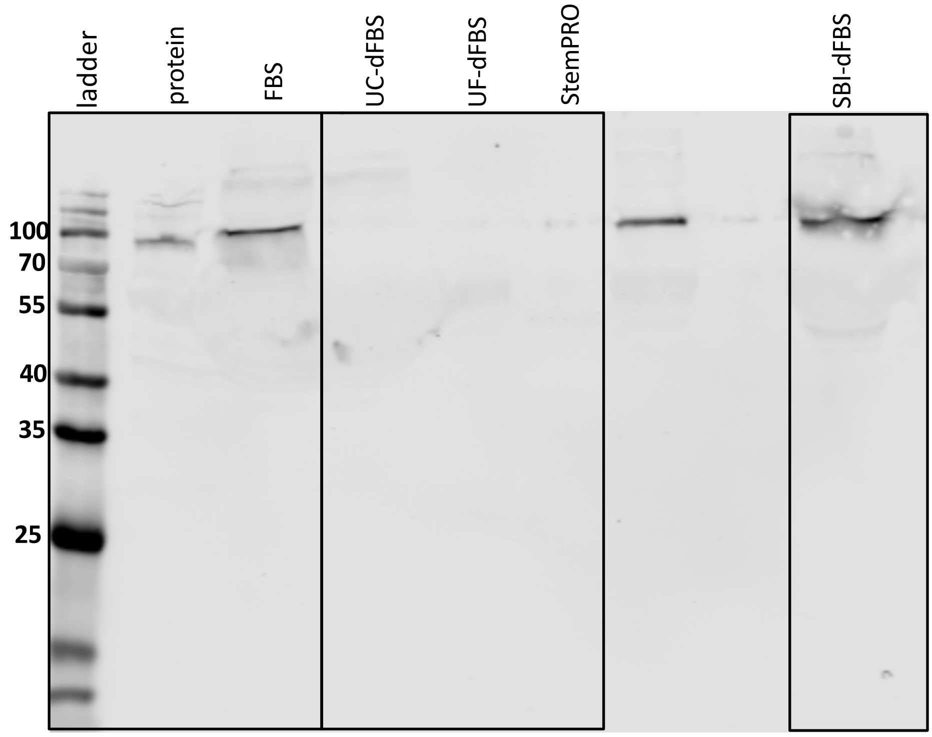

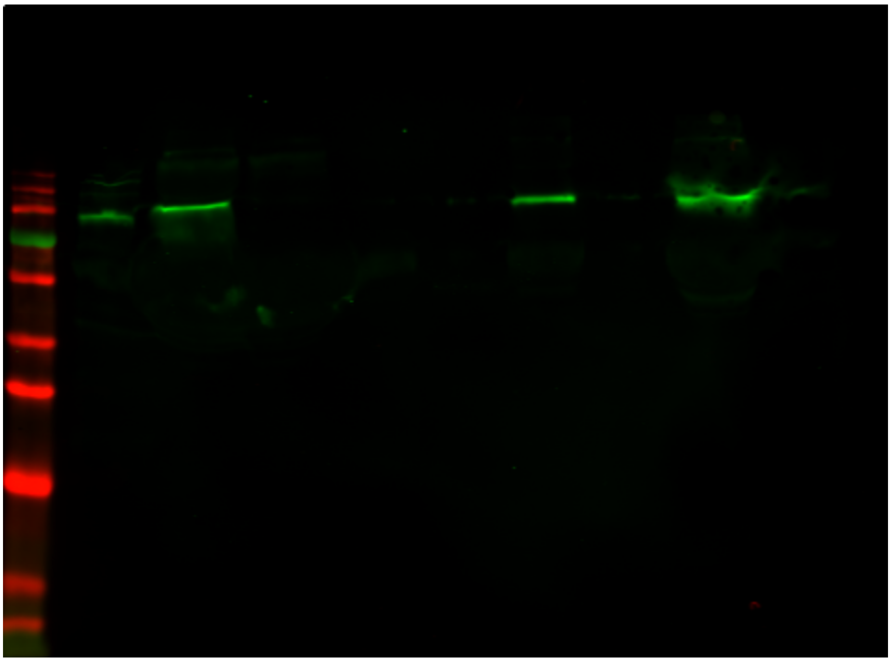

HDL

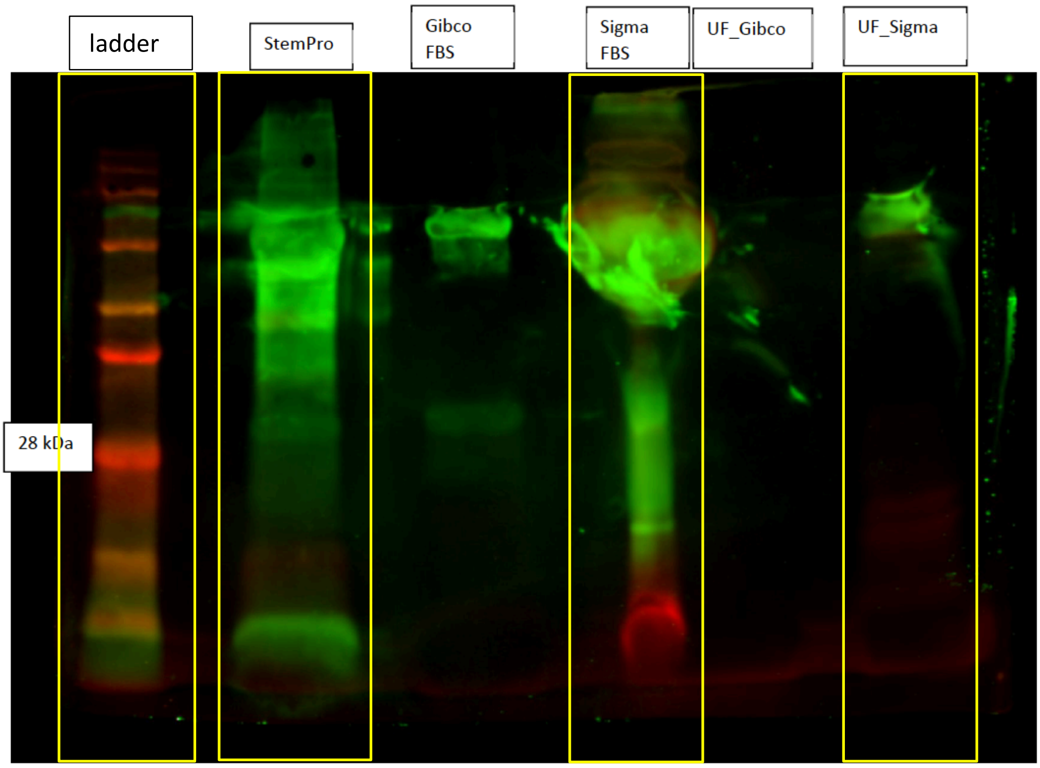
ladder
StemPro
Gibco FBS
Sigma FBS
UF_Gibco
UF_Sigma
28 kDa

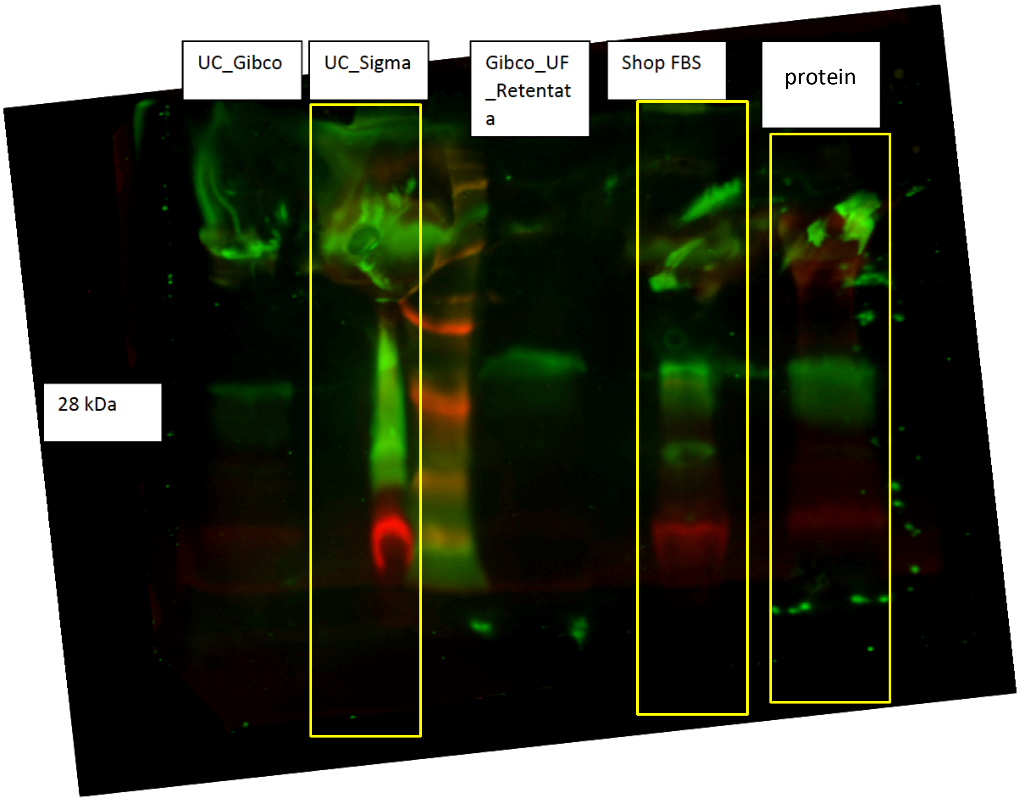
UC_Gibco
UC_Sigma
Gibco_UF_Retentata
Shop FBS
protein
28 kDa

Human HDL controls

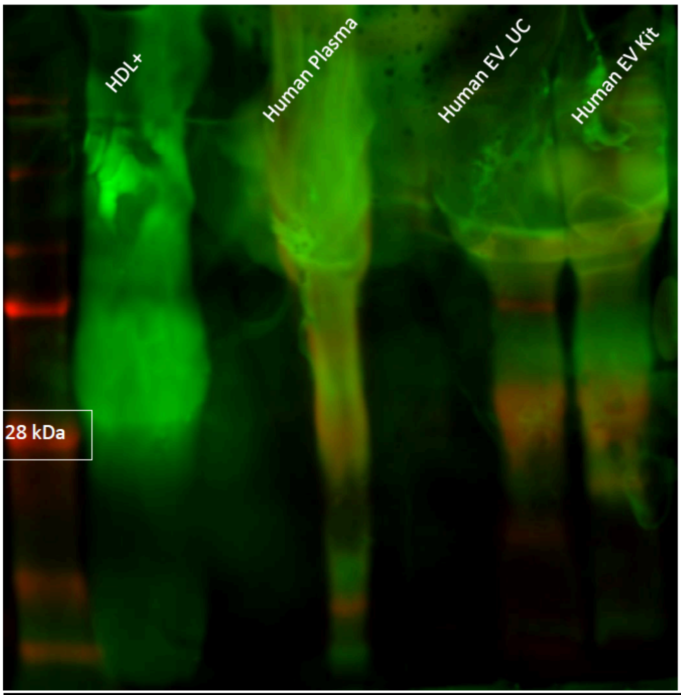

Supplement: Supplementary file 1 — Supplemental figure 1 [file 41598_2019_41772_MOESM1_ESM.pdf]
